# Prevention of peritoneal adhesions after gynecological surgery: a systematic review

**DOI:** 10.1007/s00404-024-07584-1

**Published:** 2024-06-15

**Authors:** Sebastian D. Schaefer, Ibrahim Alkatout, Nadja Dornhoefer, Joerg Herrmann, Ruediger Klapdor, Ivo Meinhold-Heerlein, Jozsef Meszaros, Alexander Mustea, Peter Oppelt, Markus Wallwiener, Bernhard Kraemer

**Affiliations:** 1 Department of Gynecology and Obstetrics, Clemenshospital Muenster, Münster, Germany; 2 Department of Gynecology and Obstetrics, University Hospital Schleswig-Holstein, Campus Kiel, Germany; 3 Department of Gynecology, University Hospital Leipzig, Leipzig, Germany; 4 Department of Gynecology and Obstetrics, Weimar Hospital, Weimar, Germany; 5 Department of Gynecology and Obstetrics, Albertinen Hospital Hamburg, Hamburg, Germany; 6 Department of Gynecology and Obstetrics, University Hospital Giessen, Giessen, Germany; 7 Department of Gynecology, Obstetrics and Reproductive Medicine, University Hospital Magdeburg, Magdeburg, Germany; 8 Department of Gynecology and Gynecological Oncology, University Hospital Bonn, Bonn, Germany; 9 Department of Gynecology, Obstetrics and Gynecological Endocrinology, Johannes Kepler University, Kepler University Hospital Linz, Linz, Austria; 10 Department of Gynecology and Obstetrics, University Hospital Halle, Halle, Germany; 11 Department of Women’s Health, University Hospital Tuebingen, Tübingen, Germany

**Keywords:** Adhesion prevention, Second-look surgery, Adhesion barrier, Peritoneal adhesions, Pelvic pain

## Abstract

**Importance:**

The formation of adhesions after gynecological surgery not only has detrimental impacts on those affected, including pain, obstruction, and infertility, but also imposes a high economic burden on healthcare systems worldwide.

**Objective:**

The aim of this review was to evaluate the adhesion prevention potential of all currently available adhesion barriers for gynecological surgery.

**Evidence acquisition:**

We systematically searched MEDLINE and CENTRAL databases for randomized controlled trials (RCTs) on the use of adhesion barriers as compared with peritoneal irrigation or no treatment in gynecological surgery. Only RCTs with second-look surgery to evaluate adhesions in the pelvic/abdominal (but not intrauterine) cavity were included.

**Results:**

We included 45 RCTs with a total of 4,120 patients examining a total of 10 unique types of barriers in second-look gynecological surgery. While RCTs on oxidized regenerated cellulose (significant improvement in 6 of 14 trials), polyethylene glycol with/without other agents (4/10), hyaluronic acid and hyaluronate + carboxymethylcellulose (7/10), icodextrin (1/3), dextran (0/3), fibrin-containing agents (1/2), expanded polytetrafluoroethylene (1/1), N,O-carboxymethylchitosan (0/1), and modified starch (1/1) overall showed inconsistent findings, results for expanded polytetrafluoroethylene, hyaluronic acid, and modified starch yielded the greatest improvements regarding adhesion reduction at 75%, 0–67%, and 85%, respectively.

**Conclusions and relevance:**

Best results for adhesion prevention were reported after applying Gore-Tex Surgical Membrane, hyaluronic acid, and 4DryField^®^. As Gore-Tex Surgical Membrane is nonabsorbable, it is associated with a greater risk of new adhesion formation due to second-look surgery to remove the product. 4DryField^®^ yielded the greatest improvement in adhesion score compared to all other barrier agents (85%). For better comparability, future studies should use standardized scores and put more emphasis on patient-reported outcome measures, such as pain and infertility.

## Introduction

### Peritoneal adhesion formation after gynecological surgery

Adhesions develop as a natural response of the peritoneum to surgical tissue trauma induced by injury (curettage), infection, radiation, ischemia, desiccation, or foreign-body reaction [1]. Indeed, intra-abdominal adhesions form after 50–100% of all abdominal surgeries [2] and after 60–90% of all gynecological procedures [3]. Tissue challenged by trauma is often characterized by decreased fibrinolytic activity, allowing fibrinous adhesions to develop into permanent, vascularized adhesions after fibroblast invasion [1]. Peritoneal adhesions frequently form after gynecological surgery, for example, ovarian cystectomy, resection of endometriosis, tubal surgery, myomectomy, and others [4]. While the formation of adhesions due to surgical intervention has been clearly established, the additional negative effect of dry CO_2_ and the intra-abdominal pressure are still being discussed controversially [5]. Peritoneal adhesions can cause great suffering in patients, mostly including pain, obstruction, and secondary infertility, as well as imposing a significant financial burden on healthcare systems [5, 6]. Surprisingly, adhesion prophylaxis has still not been adequately addressed, for example, during endometriosis surgery, and financing effective prophylaxis through healthcare systems is underrepresented although it would be crucial to achieving a satisfying outcome in the long term [7]. This might be due to scarce data on patient-reported outcomes and, therefore, a lack of data for analyzing the cost-effectiveness.

## Strategies to prevent formation of peritoneal adhesions

The exact mechanism underlying the formation of adhesions is still not completely understood. Therefore, aside from avoiding surgery, causal treatment is not available. Factors that seem to be associated with adhesion formation are use of CO_2_, level of intra-abdominal pressure, genetic factors, and traumatizing surgical techniques [8]. In addition, it has been shown in animal models that humidified CO_2_ at body temperature may prevent adhesion formation [9]. Indeed, further studies are needed to fully understand all the factors relevant to adhesion formation.

When surgery cannot be avoided, strategies for adhesion prophylaxis should include both improved surgical techniques, such as minimally invasive incisions, as well as the use of adhesion prophylactic agents and devices [7]. Laparoscopic approaches, for instance, cause less injury to the peritoneum than laparotomic approaches and therefore are associated with decreased adhesion formation [10]. Pharmaceutical approaches to prevent adhesions from developing include steroids (anti-inflammatory effect), heparin (anti-coagulatory effect), tissue-plasminogen activator (fibrinolysis), and promethazine (anti-inflammatory effect). All these molecules interfere with crucial pathways and responses during adhesion formation. Unfortunately, though, they have not been as effective as expected [11]. In contrast, the use of adhesion barriers in gynecological surgery showed promising results in randomized studies [6]. Nevertheless, the comprehensive use is not common sense due to extra costs and possible side effects.

In this review, we evaluated the effect of different barrier approaches to prevent postoperative adhesions in women after gynecological surgery using a systematic literature search of randomized controlled trials (RCTs) including second-look operations, which provide the highest level of evidence.

## Methods

We searched MEDLINE and CENTRAL (Cochrane Central Register of Controlled Trials) databases in June 2023 for RCTs on the use of agents to prevent adhesions compared with flushing or no such preventive treatment in women undergoing gynecological surgery. The body of literature addressing anti-adhesive agents is extensive and the number of commercially available barriers is increasing; however, the data often lack efficacy outcome evaluated by second-look surgery. In this review, we therefore only included RCTs with second-look surgery for adhesion evaluation. Studies on intrauterine adhesions were excluded. We employed the following search strategy for MEDLINE: (second-look OR 2nd look OR reintervention OR reoperation OR endometr* OR ovar* OR laparosc* OR laparotom* OR gynec* OR fallop* OR salping*) AND adhes* AND (randomized controlled trial[Publication Type] OR randomized[Title/Abstract] OR placebo[Title/Abstract]) and the following for CENTRAL: randomized controlled trial in Abstract AND second-look OR 2nd look OR reintervention OR reoperation OR endometr* OR ovar* OT laparosc* OR gynec* OR fallop* OR salping* in Title Abstract Keyword AND Journal article in Publication Type.

Two authors (SS and BK) independently assessed the identified literature, based on set criteria to minimize the risk of bias. Data were extracted independently by two authors (IA, IMH) according to the study protocol that was prospectively registered in PROSPERO (international prospective register of systematic reviews) with the ID CRD42023428551. Inconsistencies were identified and resolved under the direction of the lead author (SS). Results were reported according to the Preferred Reporting Items for Systematic reviews and Meta-Analyses (PRISMA) statement [12]. Similar review articles were screened to assure the completeness of the search results.

Whenever possible, American Fertility Society (AFS) adhesion scores were used as an effect measure to assess the potential of the barriers. Using AFS scores, the different barrier devices can be directly compared. When AFS scores were not available, other scores were considered. To further enhance comparability, absolute scores at second-look surgery were chosen whenever available as they are more commonly used than the change in scores between the interventions. When no score was available, other measures such as adhesion incidence were applied. The outcome measure “adhesion-free patients” was considered to have the lowest explanatory power.

## Results

**Fig. 1:**
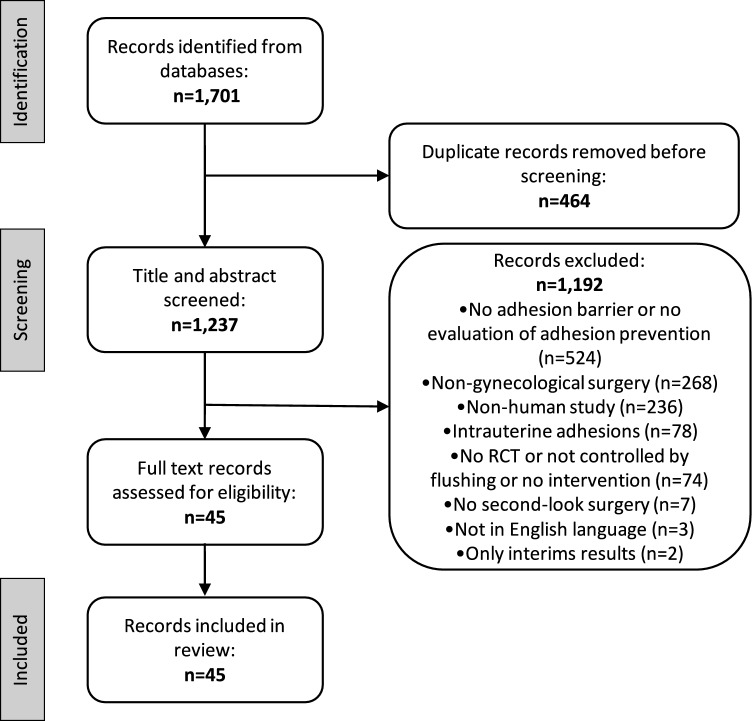
Preferred reporting items for systematic reviews and meta-analyses (PRISMA) flow diagram

The systematic search of MEDLINE and CENTRAL identified 1237 unique articles. A total of 1192 articles were excluded after reviewing the titles and abstracts for the following reasons: no adhesion barrier or no evaluation of adhesion prevention, nongynecological study, nonhuman study, intrauterine adhesions, no RCT, no second-look surgery, study not in English language, or only interim results included (Fig. 1).

### Barrier agents

The systematic search identified 10 unique types of barrier agents: oxidized regenerated cellulose (ORC) was used in 14 studies [10, 13–25], polyethylene glycol (PEG) with/without other agents in 10 studies [26–35], hyaluronic acid and hyaluronate + carboxymethylcellulose (HA/CMC) in 10 studies [36–45], icodextrin in 3 studies [46–48], 32% dextran 70 in 3 studies [49–51], fibrin-containing agents in 2 studies [52, 53], and expanded polytetrafluoroethylene (ePTFE) [54], N,O-carboxymethylchitosan (NOCC) [55] and modified starch [6] in 1 study each.

The basic function of all these products is to physically separate injured tissues during the initial healing process.

### Oxidized regenerated cellulose (ORC)

Among the different types of adhesion prevention barriers, ORC was the one studied most widely, with 14 RCTs found (Tab. 1). Regenerated cellulose is manufactured from natural cellulose sources such as wood. Interceed^®^ (Johnson & Johnson, New Brunswick, NJ, USA), the most commonly used ORC, forms a gelatinous barrier.

**Table 1:** RCTs using ORC for adhesion prevention after gynecological surgery. For scores, standard deviation or standard error of the mean are given (depending on the values provided in the original papers) if available; significant *P* values (< 0.05) are written in bold; na = not available

Study	Product name	Indication	No. of patients^a^	Time to 2nd look [weeks]	Outcome evaluation	Outcome control	Outcome intervention	Improvement^b^	*P* value	Follow-up
Tinelli et al. [10]	Interceed^®^	Intracapsular myomectomy by laparoscopy	546	na	Rate of adhesions	23%	16%	7%	Not significant	No
		Intracapsular myomectomy by laparotomy				28%	22%	6%	Not significant	
Mais et al. [21]	Interceed^®^	Myomectomy	50	12–14	Adhesion-free patients	12%	60%	48%	** < 0.05**	No
Sawada et al. [23]	Interceed^®^	Myomectomy, cystectomy, tuboplasty	23	na	Incidence of adhesions	85.7%	37.5%	48.2%	Not significant	Pregnancy rate
Keckstein et al. [18]	Interceed^®^	Ovarian cystectomy	17^c^	8–30	Adhesion-free outcome	35%	76%	41%	** < 0.05**	No
Saravelos and Li [22]	Interceed^®^	Polycystic ovarian syndrome	21^c^	2–11	Incidence of adhesions	33%	43%	-10%	Not significant	Pregnancy rate
Greenblatt and Casper [17]	Interceed^®^	Polycystic ovarian syndrome	7^c^	3–4	Score	6.71 ± 5.99	9.86 ± 10.21	-47%	0.5	Pregnancy rate
Franklin [16]	Interceed^®^	Ovarian adhesions	55^c^	1.5–14	Severity score	1.15 ± 0.91	0.84 ± 0.96	27%	0.086	No
Nordic Adhesion Prevention Study Group [14]	Interceed^®^	Adhesiolysis on ovaries, fallopian tubes, and fimbriae	66^c^	4–10	Total adhesion score	5.0 ± 0.06	2.95 ± 0.05	41%	** < 0.01**	No
Li and Cooke [19]	Interceed^®^	Adhesiolysis	28^c^	3–14	Score	0.93 ± 0.68	0.7 ± 0.61	24%	Not significant	No
Azziz [15]	Interceed^®^	Adhesiolysis	134^c^	1.5–14	Patients without adhesion re-formation	24%	51%	27%	Not significant	No
INTERCEED(TC7) Adhesion Barrier Study Group [13]	Interceed^®^	Infertility, adhesions	74^c^	1.5–14	Adhesion incidence	72%	46%	26%	**0.003**	No
Sekiba [24]	Interceed^®^	Adhesiolysis	63^c^	1.5–14	Adhesion incidence	76%	41%	35%	** < 0.0001**	No
		Infertility and endometriosis	28^c^	1.5–14		82%	50%	32%	** < 0.05**	No
Wallwie-ner et al. [25]	Interceed^®^	Endometriosis	40	13–26	Score	1.1 ± 0.91	0.4 ± 0.68	64%	0.058	No
Mais et al. [20]	Interceed^®^	Endometriosis	32	12–14	Adhesion-free patients	13%	75%	62%	** < 0.05**	No

^a^Number of patients in the primary outcome evaluation

^b^Improvement was calculated as (Control-Intervention)/Control*100 for outcomes not measured in %, and by direct subtraction for outcomes measured in %

^c^Study used intra-patient controls (one side treated with the respective product and the other side served as an untreated control); thus, all patients belong to both groups

From the 14 studies reported, 6 showed a significant improvement in adhesion formation with ORC compared to controls, while 8 did not, with 2 even showing a deterioration [17, 22].

### Polyethylene glycol (PEG) with/without other agents

PEG is a hydrophilic polymer used for adhesion prevention and is available in different forms: SprayShield^™^ (Covidien, Waltham, MA, USA, which replaced SprayGel^™^ (Confluent Surgical, Inc., Waltham,MA, USA)) and CoSeal^®^ (Angiotech Pharmaceuticals, Inc, Vancouver, Canada) are two-component systems consisting of two different PEG solutions. SprayShield^™^ was developed on the basis of Spraygel^™^ and has a shorter absorption time that is supposed to reduce foreign-body reaction [56]. Oxiplex/AP Gel (FzioMed, Inc., San Luis Obispo, California, USA, alias Intercoat^®^) is a gel composed of PEG and CMC. PREVADH^™^ (Covidien, Trévoux, France) is a more recently studied barrier material consisting of porcine collagen, PEG, and glycerol that forms a hydrogel when hydrated [26]. The latest PEG-containing adhesion barrier is ACTAMAX^™^, a combination of two aqueous solutions of dextran aldehyde and PEG amine polymers, which form a hydrogel when mixed [34]. We found 10 RCTs using PEG-based barriers for adhesion prevention (Tab. 2).

**Table 2:** RCTs using PEG or PEG combinations for adhesion prevention after gynecological surgery

Study	Product name	Indication	No. of patients^a^	Time to 2nd look [weeks]	Outcome evaluation	Outcome control	Outcome intervention	Improvement^b^	*P* value	Follow-up
Trew et al. [34]	ACTAMAX^™^	Gynecologic surgery	63	4–12	Score	2.32 ± 1.48	1.83 ± 1.5	21%	0.228	No
Canis et al. [26]	PREVADH^™^	Myomectomy	54	10–20	Score (mAFS)	1.2	0.8	33%	0.23	Pregnancy rate
Tchartchian et al. [32]	SprayShield^™^	Myomectomy	13	8–12	Severity score	1.63 ± 1.06	0.8 ± 1.1	51%	0.205	No
					Extend score	0.92 ± 0.66	0.6 ± 0.89	35%	0.477	
Ten Broek et al. [33]	SprayGel^™^	Adhesiolysis, salpingotomy and/or cystectomy	15	4 (mean)	Score	2.4 ± 2.4	1.2 ± 1.3	50%	0.29	No
Mettler et al. [30]	SprayGel^™^	Myomectomy	40	3–16	Severity Score	1.9	1.0	47%	**0.002**	No
Johns et al. [28]	SprayGel^™^	Ovarian Surgery	14^c^	3–16	Severity score	2.7	2.1	22%	Not significant	No
Mettler et al. [31]	CoSeal^®^	Myomectomy	58	8–10	Score (mAFS)	2.6 ± 2.2	1.1 ± 1.9	58%	**0.02**	No
diZerega et al. [27]	Oxiplex/AP Gel	Adnexal surgeries	37	6–10	Score (AFS)	14.0 ± 3.0	6.2 ± 2.0	56%	**0.01**	No
Young et al. [35]	Oxiplex/AP Gel	Adnexal surgery	28	6–10	Score (AFS)	11.6	8.1	30%	Not significant	No
Lundorff et al. [29]	Oxiplex/AP Gel	Adnexal surgeries	49	6–10	Score (AFS)	15.8	9.1	42%	** < 0.01**	No

(m)AFS, (modified) American Fertility Society

^a^Number of patients in the primary outcome evaluation

^b^Improvement was calculated as (Control-Intervention)/Control × 100 for outcomes not measured in %, and by direct subtraction for outcomes measured in %

^c^Study used intra-patient controls (one side treated with the respective product and the other side served as an untreated control); thus, all patients belong to both groups

Bold marks significant p values

SprayGel^®^ significantly improved adhesion scores in 1 of 3 RCTs identified. The study evaluating CoSeal^®^ reached significance but lacked investigator blinding and the evidence level is therefore considered lower than that from the other studies [31]. Results of the study using SprayShield™ did not reach statistical significance. However, they showed a positive trend. Oxiplex/AP gel was used in 3 RCTs: 2 of them found a significant improvement in AFS score compared to controls. The latest RCTs on the use of PEG with PREVADH^™^ and ACTAMAX^™^ did not show PEG to be superior to control treatment; however, ACTAMAX^™^ achieved a significantly greater change in score between both surgeries vs. in controls [34].

### Hyaluronic acid (HA) / Hyaluronate with or without carboxymethyl cellulose (HA/CMC)

Hyaluronic acid (HA) is a natural component of the extracellular matrix that is also present in the peritoneal fluid. HA has been being used for adhesion prevention for over 10 years under the brand names HyaRegen^®^ (BioRegen Biomedical, Changzhou, China), Hyalobarrier^®^ (auto-crosslinked HA, Fidia Advanced Biopolymers, Abano Terme, Padova, Italy), Intergel^®^ (ionically cross-linked HA, discontinued, Lifecore Biomedical, Inc., Chaska, MN, USA), and Sepracoat (unmodified HA, discontinued, Genzyme Corporation, Cambridge, MA, USA). The combination of hyaluronate and CMC for adhesion prevention has also been studied (Seprafilm^®^ and the discontinued Sepraspray^®^, Genzyme Biosurgery, Cambridge, MA, USA). Seprafilm^®^ (Baxter Healthcare, Deerfield, IL, USA) is modified chemically and transforms into a gel after being placed on the peritoneum. HA- and HA/CMC-based products were used in 10 RCTs for adhesion prevention (Tab. 3).

**Table 3:** RCTs using HA or HA/CMC for adhesion prevention after gynecological surgery

Study	Product name	Indication	No. of patients^a^	Time to 2nd look [weeks]	Outcome evaluation	Outcome control	Outcome intervention	Improvement^b^	*P* value	Follow-up
HA										
Liu et al. [41]	Hyaregen^®^	Adhesiolysis, myomectomy, ovarian cysts, endometriosis	215	9	Score (mAFS)	0.9	0.3	67%	**0.001**	No
Mais et al. [43]	Hyalobarrier^®^	Myomectomy	43	12–14	Score	2.1 ± 2.2	2.1 ± 3.9	0%	Not significant	No
Pellicano et al. [44]	Autocrosslinked HA gel	Myomectomy	36	9–13	Patients with adhesions	77.8%	27.8%	50%	** < 0.01**	Pregnancy rate
Lundorff et al. [42]	Intergel^®^	Peritoneal cavity surgery	77	6–12	Score (mAFS)	1.25 ± 0.01	0.46 ± 0.01	63%	**0.026**	No
Johns et al. [39]	Intergel^®^	Peritoneal cavity surgery	265	6–12	Score (mAFS)	2.33 ± 2.7	1.28 ± 1.55	45%	** < 0.001**	No
Thornton et al. [45]	Intergel^®^	Peritoneal cavity surgery	23	4–12	Score (AFS)	Not specified	Not specified	Not specified	** < 0.01**	No
Diamond [37]	Sepracoat	Gynecological surgeries	245	1–32	Median de novo severity score	0.32 ± 0.07	0.2 ± 0.06	38%	**0.026**	No
HA/CMC										
Kiefer et al. [40]	Seprafilm^®^	Cesarean section	172	na	Median Adhesion Score	2	2	0%	0.647	No
Diamond [36]	Seprafilm^®^	Myomectomy	127	1–10	Score	2.43 ± 0.1 (severity) 1.68 ± 0.1 (extent)	1.94 ± 0.14 (severity) 1.23 ± 0.12 (extent)	20% (severity) 27% (extent)	** < 0.01**	No
Fossum et al. [38]	Sepraspray^®^	Myomectomy	41	4–12	Change of score (mAFS)	1.56	0.68	56%	Not significant	No

mAFS, modified American Fertility Society

^a^Number of patients in the primary outcome evaluation

^b^Improvement was calculated as (Control-Intervention)/Control × 100 for outcomes not measured in %, and by direct subtraction for outcomes measured in %

Bold marks significant p values

Of the 10 RCTs identified, 7 showed efficacy of HA or HA/CMC; 2 of the 3 studies on HA/CMC did not show any significant improvement. All but one of the studies assessed adhesion scores. Thornton et al. used adhesion scores to evaluate adhesion formation; however, they only displayed the results graphically and not numerically. Therefore, it was not possible to include score values in our analysis [45]. Separately published follow-up results of the myomectomy study by Pellicano et al. [44] further showed a significantly higher pregnancy rate after 6 and 12 (the latter after ovulation induction) months [57].

### Icodextrin.

Adept^®^ (Baxter Healthcare, Deerfield, IL, USA) is a nonviscous, iso-osmotic, clear 4% icodextrin solution that is approved for gynecological laparoscopic adhesiolysis surgery in the USA and Europe. Physical wound separation is achieved by hydroflotation. Adept^®^ was used in 3 RCTs for adhesion prevention (Tab. 4).

**Table 4:** RCTs using icodextrin for adhesion prevention after gynecological surgery

Study	Product name	Indication	No. of patients^a^	Time to 2nd look [weeks]	Outcome evaluation	Outcome control	Outcome intervention	Improvement^b^	*P* value	Follow-up
Trew et al. [46]	Adept^®^	Myomas, endometriosis	330	3–51	Score (mAFS)	8.42 ± 11.8	8.13 ± 12.37	3%	0.911	No
Brown et al. [47]	Adept^®^	Adhesiolysis	402	4–8	“Clinical success”	38%	49%	11%	**0.018**	No
diZerega et al. [48]	Adept^®^	Laparoscopic adnexal surgery	42	6–12	Score (mAFS)	0.56 ± 0.72	0.81 ± 1.42	− 45%	Not significant	No

mAFS, modified American Fertility Society

^a^Number of patients in the primary outcome evaluation

^b^Improvement was calculated as (Control-Intervention)/Control × 100 for outcomes not measured in %, and by direct subtraction for outcomes measured in %

Bold marks significant p values

Of the 3 RCTs identified, 1 showed a significant improvement, though not in the AFS scores—which were not provided—but rather in clinical success, defined as the percentage of patients in whom the number of sites with adhesions decreased by at least three or 30% of the number of sites analyzed [47]. Two studies reported adverse events, such as postoperative pain and headache [46, 48], nausea, vomiting, wound infection and vulval/genital edema [46], as well as a 45% increase in adhesion scores in the intervention group.

### Expanded polytetrafluoroethylene (ePTFE)

ePTFE is a synthetic, nonresorbable barrier agent. We found 1 study using ePTFE (Gore-Tex Surgical Membrane, W.L. Gore and Associates, Inc., Flagstaff, AZ, USA) (Tab. 5).

**Table 5:** RCT using ePTFE for adhesion prevention after gynecological surgery

Study	Product name	Indication	No. of patients^a^	Time to 2nd look [weeks]	Outcome evaluation	Outcome control	Outcome intervention	Improvement^b^	P value	Follow -up
The Myomectomy Adhesion Multicenter Study Group [54]	Gore-Tex Surgical Membrane	Myomectomy	27	2–6	Adhesion score	7.55 ± 0.57	1.88 ± 0.46	75%	** < 0.0001**	No

^a^Number of patients in the primary outcome evaluation

^b^Improvement was calculated as (Control-Intervention)/Control × 100 for outcomes not measured in %, and by direct subtraction for outcomes measured in %

Bold marks significant p values

The study identified showed a significant improvement of 75% in mean adhesion score.

### Modified starch

Starch can be modified and crosslinked to improve its capabilities in medical applications. For adhesion prevention, modified starch powder is mixed with saline to form a gel. We identified one RCT using the modified starch 4DryField^®^ for adhesion prevention (Tab. 6).

**Table 6:** RCT using modified starch for adhesion prevention after gynecological surgery

Study	Product name	Indication	No. of patients^a^	Time to 2nd look [weeks]	Outcome evaluation	Outcome control	Outcome intervention	Improvement^b^	*P* value	Follow-up
Krämer et al. [6]	4DryField^®^	Endometriosis	50	3–16	Total adhesion Score	14.2 ± 18.9	2.2 ± 3.1	85%	**0.004**	Pregnancy rate, pain scores

^a^Number of patients in the primary outcome evaluation

^b^Improvement was calculated as (Control-Intervention)/Control × 100 for outcomes not measured in %, and by direct subtraction for outcomes measured in %

Bold marks significant p values

4DryField^®^ reached a significant improvement of 85% in mean total adhesion score compared to peritoneal irrigation with saline. Total adhesion score was calculated by multiplication of the extent and severity score for the evaluated sites followed by summing up over all sites. Separately published follow-up results of this RCT further showed that fertility was significantly higher and pain scores reduced [58].

### Other approaches

Three other types of adhesion barriers were examined in RCTs between 1983 and 2011, but none of them have been the subject of recently conducted trials: (1) dextran, a chain polysaccharide derivative of sugar beets, which is a very viscous agent that is very slowly absorbed from the peritoneal cavity; (2) gelatinous fibrin formed from fibrinogen and thrombin, which remains on the spot as a stable barrier for about 1 week. While impaired fibrinolysis has been identified as a factor increasing the probability of adhesion formation, the application of fibrin-containing gel or sheets—at least in theory—act as an adhesion preventive barrier agent as it directly closes wound surfaces, decreasing the physiological cascade of adhesion formation during wound-healing; (3) N,O–carboxymethylchitosan (NOCC), a purified derivative of chitin obtained from shrimp, which is similar in structure to HA and CMC. We identified three RCTs using 32% dextran 70 (Hyskon^®^, Hyskon, Pharmacia, Inc., Piscataway, NJ, USA) for adhesion prevention, two studies with fibrin barrier agents (Adhexil^™^ (Omrix Biopharmaceuticals, Inc., Ness Ziona, Israel) and fibrin gel/sheet), and one RCT using NOCC (Tab. 7).

**Table 7:** RCTs using 32% dextran 70, fibrin or N,O-carboxymethylchitosan for adhesion prevention after gynecological surgery

Study	Product name	Indication	No. of patients^a^	Time to 2nd look [weeks]	,Outcome evaluation	Outcome control	Outcome intervention	Improvement^b^	*P* value	Follow -up
Rosenberg and Board [51]	Hyskon^®^	Infertility	44	2–17	Adhesion score	7.38	5.39	27%	Not specified	No
Larsson et al. [50]	Hyskon^®^	Tubal surgery	105	4–10	Adhesion score	9.93 ± 9.28	10.71 ± 8.93	− 8%	Not significant	Pregnancy rate
Adhesion Study Group [49]	Hyskon^®^	Distal tubal disease, endometriosis, or pelvic adhesions	91	8–12	Adhesion Score	4.83 ± 0.91	3.63 ± 0.81	25%	Not significant	No
Diamond et al. [52]	Adhexil^™^	Ovarian surgery	16^c^	3–10	mAFS score (ovary only)	7.1 ± 6.9	4.6 ± 6.9	35%	0.38	No
Takeuchi et al. [53]	Fibrin gel	Myomectomy	91	8–32	Incidence of adhesions	62.5%	34.5%	28%	** < 0.05**	No
	Fibrin sheet						67.7%	− 5.2%	Not significant	
Diamond et al. [55]	N,O-carboxy-methyl-chitosan	Gynecological practices	32	2–10	Severity score	0.54	0.7	− 30%	Not specified	No
					Extend score	0.26	0.39	− 50%	Not specified	

^a^Number of patients in the primary outcome evaluation

^b^Improvement was calculated as (Control-Intervention)/Control × 100 for outcomes not measured in %, and by direct subtraction for outcomes measured in %

^c^Study used intra-patient controls (one side treated with the respective product and the other side served as an untreated control); thus, all patients belong to both groups

Bold marks significant p values

Of the three RCTs studying 32% dextran 70, those of Larson et al. and Rosenberg and Board showed improvements in net changes of adhesion scores, which in the latter case was statistically significant [50, 51]. Nevertheless, Rosenberg and Board did not provide a statistical comparison of adhesion scores [51]. Of the two RCTs studying fibrin, treatment with Adhexil^™^ showed a trend toward improved adhesion prevention (mAFS); however, the difference was not statistically significant [52]. Takeuchi et al. compared fibrin sheet or fibrin gel with a control group and found that fibrin gel significantly reduced the incidence of adhesions; however, no score was assessed [53]. In their RCT on NOCC, Diamond et al. found an increase in adhesion scores in the intervention group at second look vs. controls [55]. When these were offset against the scores from the first surgery, both results were better for the intervention group, albeit not significantly.

## Discussion

A strategy for preventing adhesion formation during gynecological surgery is crucial to avoiding complications and has the potential to spare resources and thus save costs in healthcare systems [8]. A promising and widely studied strategy is the use of barrier agents. Before adhesion barriers can be used routinely, however, their ability to prevent the formation of adhesions must be thoroughly tested in the respective context. Furthermore, potential side effects of resorbable and of nonresorbable agents need to be evaluated and compared to the appropriateness of the expected positive effects with the possible toxic risks. Various barriers have been used to prevent adhesions and numerous studies have been published in the field of gynecological surgery. The assessment of adhesions in second-look surgery varies between the individual studies. Therefore, a comparative efficacy evaluation of the barriers is challenging. To address this issue, we looked for adhesion scores as primary outcome measure. When scores were not available, we focused on the incidence of adhesions and, finally, adhesion-free patients, the latter being considered as the lowest explanatory quality. As most studies provided adhesion scores, informed judgments of the effectiveness of the respective barrier devices were feasible. Generally, an ideal barrier should exert its effect on the wound at least until the wound-healing processes are complete, which takes about 5–6 days postoperatively [59–61]. In a recent article on the topic, a time of about 7 days is suggested to be ideal [8]. Nevertheless, further research is needed to elucidate the exact timing of wound-healing processes and, consequentially, the optimal breakdown times for absorbable barrier agents. In addition, the ideal adhesion barrier should not have to be removed again, interfere with peritoneal healing, or be influenced by the presence of blood [36]. In summary, an ideal barrier agent should be biodegradable, laparoscopically applicable, clinically efficacious, and affordable for daily routine use [8].

The potential of adhesion prevention with icodextrin is not convincing as only 1 of 3 RCTs showed a significant improvement by 11% compared to controls [47]. The outcome measure here was “clinical success”, whereas it was mAFS in the other 2 studies. Adept^®^ did not significantly reduce adhesions in several preclinical trials either [62–66]. Icodextrin solution is rapidly absorbed from the abdominal cavity and, thus, the retention time of about 7 days required for optimal adhesion prevention due to peritoneal healing is not achieved. This could potentially explain why Adept^®^ delivered results inferior to those for (semi)solid barriers. Additional drawbacks of Adept^®^ are the very narrow indication (it is only intended for gynecological laparoscopic adhesiolysis surgery) and the lacking possibility to use drains. Furthermore, Lee et al. [67] showed that using 4% icodextrin the incidence of small bowel obstruction was higher than when not using any anti-adhesion materials [27], and severe small bowel serosal fibrosis and dense adhesions were also reported in patients in whom 4% icodextrin was used in abdominal surgery [68].

The 3 RCTs using 32% dextran 70 (Hyskon^®^) for adhesion prevention did not yield significant improvements in adhesion scores; however, one study showed significant improvement in net adhesion scores [51] and one in adhesion scores of patients with a severe initial extent of adnexal adhesions [49]. The studies were conducted in 1983–1985, though, and no further RCTs have been published since then as safety concerns were reported regarding the proliferation of bacteria in Hyskon^®^-containing media and concerning the anaphylactoid potential reported [49].

Adhesion prevention with fibrin was assessed in 2 RCTs, with only one presenting mAFS scores. The latter study showed a trend toward successful adhesion prevention with Adhexil^™^ but only included a small population of 16 patients [52]. The other study found a significant reduction in the incidence of adhesions in the fibrin gel group, but no improvement with fibrin sheets [53]. As no score was assessed in the second RCT, no sound comparison of the effectiveness of fibrin in these 2 studies can be made. Although fibrin has important characteristics of an ideal barrier [69], such as safety and remaining on the surgical site for about 1 week, its efficacy is questionable based on the data presently available.

ePTFE-based Gore-Tex Surgical Membrane achieved a significant improvement in mean adhesion score in the RCT found [54]. In a comparative study without a control group not included in the present review, ePTFE was found to be superior to ORC (Interceed^®^) in preventing adhesions after adnexal surgery [70]. However, since the Gore-Tex Surgical Membrane has to be removed again as it is nonresorbable, ePTFE cannot be recommended for preventing adhesions due to the requirement of a subsequent surgical intervention, imposing once again the risk of adhesion formation, in addition to the other risks and complications that naturally occur during and after surgeries. ePTFE, therefore, plays no role in today’s surgical routine in gynecology.

NOCC was used in 1 RCT where severity and extent of adhesions at second look were even higher in the intervention group. Although a change in adhesion score and number of sites with recurrent adhesions were reduced, these results were not statistically significant [55]. NOCC does not mix easily with blood, a desired feature of an adhesion barrier. As this was the pilot clinical study using NOCC and no further RCTs were conducted, it is not possible to reach a valid conclusion regarding the effectiveness of NOCC in preventing adhesions in humans.

ORC (Interceed^®^) was the most widely studied barrier agent, with 14 RCTs identified; however, the adhesion prevention potential was unconvincing: 6 RCTs showed a significant improvement in adhesion prevention with ORC compared to controls [13, 14, 18, 20, 21, 24]. In only one of these trials was a more than 50% improvement achieved [20], indicating that a large proportion of adhesions remained. Furthermore, the explanatory power of most outcome measures is considered rather low (adhesion-free patients, adhesion incidence) and 2 studies even showed a trend toward elevated adhesion formation after applying Interceed^®^.[17, 22] The authors explained these results with the assumed differential potential of Interceed^®^ in reducing de novo or reformed adhesions and because the whole ovary was not covered with the barrier. Similarly, unconvincing results have been reported in numerous preclinical studies in which Interceed^®^ failed to prevent adhesion formation [65, 71–76]. Another drawback of this adhesion barrier is its reported incompatibility with the presence of blood [8] as this would lead to adhesion formation through the barrier [77, 78] and adhesions that formed despite the presence of Interceed^®^ were histologically shown to include substantial amounts of product remnants at agglutination sites associated with a local inflammatory response [65]. Accordingly, it would be almost impossible to use this product after myomectomy considering that hemostasis at the myomectomy site is rarely complete [54]. Preclinical studies further showed an inflammatory response, as well as sloughing of intact peritoneum when ORC was applied, inducing de novo adhesion formation [65, 79].

PEG (SprayShield^™^, SprayGel^™^, Oxiplex/AP Gel alias Intercoat^®^, PREVADH^™^, and ACTAMAX^™^) was used in 10 RCTs. All 10 RCTS used adhesion scores and therefore exhibit high explanatory power. SprayGel^®^ showed significant improvements in adhesion prevention in 1 of 3 RCTs identified, exhibiting a 58% improvement [80]. SprayShield^™^ was used in one study in 2014 and the results did not reach statistical significance. However, they showed a trend toward a positive effect of SprayShield^™^ [32]. In 2 of the 3 RCTs on Oxiplex/AP gel, a significant improvement in AFS score was shown compared to controls, while in the third, in which patients primarily received adhesiolysis, the score in the intervention group was identical for both the second and first surgeries. Furthermore, two RCTs examining Oxiplex/AP Gel for intrauterine adhesion prevention also found contradictory results [81, 82]. A general drawback of this product could be its long resorption time, which might induce foreign-body reactions. PREVADH^™^ did not significantly improve the mAFS. ACTAMAX^™^ did not lead to improved adhesion scores either but did achieve a significantly greater change in score than in controls. The authors noted that the ACTAMAX^™^ study was not powered to detect differences between randomized groups in efficacy outcomes. In summary, proof of efficacy of PEG-based barriers is inconclusive. The simple application and the possibility of covering large areas is an advantage of SprayShield^™^; however, its longer resorption time could possibly result in foreign-body reactions. The rather high costs of SprayGel^™^ are also a drawback of this specific PEG adhesion barrier [4].

Of the 10 RCTs identified using HA (HyaRegen^®^, Hyalobarrier^®^, Intergel^®^ and Sepracoat) or HA/CMC (Seprafilm^®^ and Sepraspray^®^), 7 showed superiority compared to controls, including 6 of 7 studies on HA and 1 of 3 studies on HA/CMC. All but one of the studies assessed adhesion scores. Liu et al. showed the greatest effect with HA with an improvement of 67% in mAFS score using the auto-crosslinked HyaRegen^®^ gel [41]. However, the other auto-crosslinked HA barrier, Hyalobarrier^®^, showed no improvement at all [43] and the third trial using an auto-crosslinked HA barrier did not report adhesion scores. Accordingly, proof for efficacy of this material remains inconclusive. This is further reinforced by results of several trials examining intrauterine adhesion prevention with auto-crosslinked HA gels, which also achieved contradictory results [83–86], as well as preclinical studies which found that it was not effective [87–89]. HA-based barrier Intergel^®^ was associated with safety issues in an abdominal surgery RCT concerning postoperative morbidities, particularly causing peritonitis, anastomotic dehiscence, and prolonged ileus [90]. This product has been discontinued. Furthermore, there are safety concerns based on in vitro data demonstrating that HA may promote tumor growth [91] although a clinical evaluation seems to dispel these doubts at least for the HA/CMC combination product Seprafilm^®^.[92] As Sepraspray^®^ has been discontinued, the results for Seprafilm^®^ are of greater interest concerning HA/CMC combinations. The most recent study on Seprafilm^®^ did not show any difference in the mean adhesion score [40] and therefore could not confirm the significant improvements reported by Diamond in 1996 [36]. In several preclinical studies, it also failed to prevent adhesion formation [65, 75, 89]. Major drawbacks of Seprafilm^®^ are that it cannot be applied easily laparoscopically yet and that it can be difficult to adequately cover uneven surfaces [36]. Furthermore, a large RCT in abdominal surgery found that Seprafilm^®^ significantly increases the incidence of fistulas, peritonitis, and anastomotic leakage [93]. A significantly higher risk of anastomotic leak was confirmed based on data from eight studies and 3,037 patients [94]. Therefore, the routine use of HA or HA/CMC is not supported by the present data and further studies should be performed.

The modified starch-based device 4DryField^®^ yielded a significant improvement, reaching a mean total adhesion score reduction of 85% in its first RCT [6]—the best result achieved with an absorbable barrier. In this RCT, patients with deep infiltrating endometriosis were included, who received excision for histological confirmation and symptom relief during the first and complete excision during the second intervention, where adhesion scoring was performed by an assessor blinded to the patient’s group assignment. The outcome is in line with results from non-RCTs: 85% after endometrioma resection [95], up to 75% after gynecological adhesiolysis [96], 87.5% after release of adhesive small bowel obstruction [97], and 100% in preclinical trials [98]. Furthermore, a preclinical trial comprising a direct comparison with Interceed^®^ (ORC), Seprafilm^®^ (HA/CMC), and Adept^®^ (icodextrin) showed that 4DryField^®^ is significantly more effective than any of these products [65]. Adhesion reduction was 93% with 4DryField^®^, 54% with Seprafilm^®^, 16% with Adept^®^ and 4% with Interceed^®^. This trial also compared the two possible ways of application for 4DryField^®^. Applied as powder and subsequently transformed into a gel by dripping, adhesion reduction was 100%, while it was 85% when applied directly as an *ex situ* premixed gel (difference between these two ways of application not statistically significant). Additional preclinical and clinical trials showed that other currently available modified starch powder-based agents did not yield a statistically significant reduction in adhesion formation.[98–100]. It has been supposed that one factor contributing to these results is the fact that all of these exhibit retention times of only up to three days maximum and, therefore, are absorbed before mesothelial healing is completed [8, 100].

In general, patient-reported outcome measures are of high clinical relevance when considering the use of adhesion barriers. Despite the importance of such data, a 2020 Cochrane Review on barrier agents did not find any studies examining the influence on live birth rates or pelvic pain [101]. In addition, only seven of the 45 RCTs included in the present review article presented follow-up results for fertility and/or pelvic pain. Of these seven RCTs, all reported on pregnancy rates, but only one examined postoperative pain development. Three of the six RCTs examined pregnancy rates after application of Interceed^®^[17, 22, 23]. It is not possible to draw any conclusions on the influence of Interceed^®^ for the studies by Greenblatt and Casper [17] and Saravelos and Li [22]. However, as in all patients, the product was applied to one ovary, while the other was left untreated and served as an internal control. In both studies, adhesion scores at the ovaries treated with Interceed^®^ were higher than those of the control ovaries, showing statistically nonsignificant deteriorations [17, 22]. The third study on Interceed^®^ did not reach a significant improvement in the incidence of adhesions but found an increased pregnancy rate after 2 years (78.3% vs. 46.7%, *P* < 0.049) [23]. Of the remaining four studies dealing with patient-reported outcome measures, one examined PREVADH^™^, one Hyskon^®^, one Hyalobarrier^®^, and one 4DryField^®^. The study on PREVADH^™^ did not yield a significant improvement in adhesion scores but did find an increased pregnancy rate after 3 years (64% vs. 24%, *P*  = 0.02) [26]. The study on Hyskon^®^ showed nonsignificant deteriorations in both adhesion scores (9.93 vs. 10.71) and pregnancy rate (23.5% vs. 33.3%) in the intervention group [50]. The studies on Hyalobarrier^®^ and 4DryField^®^ are the only RCTs achieving significant improvements in both adhesion scores and pregnancy rates in the intervention group [6, 57, 58]. After 12 months, pregnancy rate was 39% after myomectomy and 21% after endometriosis resection when no adhesion prevention was utilized. These rates could be doubled from 39 to 78% when Hyalobarrier^®^ was applied and tripled from 21 to 64% when 4DryField^®^ was used. In the study on Hyalobarrier^®^, patients who did not conceive after 6 months underwent ovulation induction from the 7th to the 12th follow-up month [57], while in the study on 4DryField^®^ patients did not undergo ovulation induction. As several studies have described adhesions as the main cause of secondary infertility, it is conceivable that the higher pregnancy rates are related to reduced adhesion formation [58]. The 4DryField^®^ study is the only RCT examining the influence of an adhesion barrier on pain relief. In the intervention group, all of the five pain scores examined [cycle-independent pelvic pain (CIPP), dysmenorrhea, dyspareunia, dyschezia, and dysuria] were lower than in the control group 12 months after the second intervention [58]. Furthermore, the scores for CIPP, dysmenorrhea, and dyspareunia were significantly lower than before the first surgery in the intervention group, while only dysmenorrhea was significantly reduced in the control group [58]. As a correlation between adhesion formation and pain has been shown in many studies, it is conceivable that the more favorable pain development in the intervention group is linked to an effective prevention of adhesion formation in this group as compared to the control group [58]. Furthermore, patients in the control group had increasing pain scores in the later course of the follow-up period, which might be explained by the general presence of adhesions and an increasing adhesion severity over time being causative for pain recurrence [58]. The intervention group, in contrast, did not show an increase in pain scores and generally exhibited more favorable pain score results, likely due to the reduction of adhesion formation by the adhesion barrier [58]. Considering the high relevance of follow-up results, particularly for patients but also for surgeons, hospitals, and the healthcare system, the very limited availability of such data from RCTs is hardly understandable and constitutes a severe gap in scientific knowledge. Therefore, it is urgently recommended to include follow-up data and patient-reported outcome measures in future RCTs.

## Conclusion

It is crucial in gynecological surgery to prevent adhesions and protect women from pain, infertility, bowel obstructions, and need for repeated operations. The most promising adhesion score reductions, the outcome measure of the highest explanatory quality, were achieved using Gore-Tex Surgical Membrane with 75%, hyaluronic acid with 0–67%, and 4DryField^®^ with 85% improvement. As Gore-Tex Surgical Membrane is nonabsorbable, it must be removed again surgically, imposing a greater risk of complications and, consequentially, adhesion formation in contrast to HA and 4DryField^®^. 4DryField^®^, which is associated with the most pronounced improvement in adhesion scores of all the barrier agents, combines a resorption time of about 7 days with ease of use and a favorable safety profile. Future studies should use standardized scores such as the mAFS to generally facilitate comparability of the results. Furthermore, patient-reported outcome measures, such as pain and infertility, should be emphasized more in future trials, and corresponding endpoints should be collected. If these aspects are considered, it could become easier to address the multiple and diffuse risks arising from postoperative adhesion formation. The authors’ vision that iatrogenically caused adhesions can be minimized due to a general use of agents for adhesion prevention that are not associated with any risk seems achievable.

## Data Availability

Data will be made available upon reasonable request by the authors.
